# VertexWiseR: A package for simplified vertex-wise analyses of whole-brain and hippocampal surfaces in R

**DOI:** 10.1162/imag_a_00372

**Published:** 2024-11-14

**Authors:** Charly Hugo Alexandre Billaud, Junhong Yu

**Affiliations:** Nanyang Technological University, Psychology, School of Social Sciences, Singapore, Singapore

**Keywords:** package, R, cortical surface, TFCE, linear models, hippocampus, FreeSurfer, vertex-wise

## Abstract

Currently, whole-brain vertex-wise analyses on brain surfaces commonly require specially configured operating systems/environments to run and are largely inaccessible to R users. As such, these analyses are inconvenient to execute and inaccessible to many aspiring researchers. To address these limitations, we present VertexWiseR, a user-friendly R package, to run cortical and hippocampal surface vertex-wise analyses, in just about any computer, requiring minimal technical expertise and computational resources. The package allows cohort-wise anatomical surface data to be highly compressed into a single, compact, easy-to-share file. Users can then run a range of vertex-wise statistical analyses with that single file without requiring a special operating system/environment and direct access to the preprocessed file directories. This enables the user to easily take the analyses “offline”, which would be highly appropriate and conducive in classroom settings. This R package includes a conventional suite of tools for extracting, manipulating, analyzing, and visualizing vertex-wise data, and is designed to be easy for beginners to use. Furthermore, it also contains novel or advanced functionalities such as hippocampal surface analyses, meta-analytic decoding, threshold-free cluster enhancement, and mixed-effects models that would appeal to experienced researchers as well. In the current report, we showcase these functionalities in the analyses of two publicly accessible datasets. Overall, our R package opens up new frontiers for the R’s user base/community and makes such neuroimaging analyses accessible to the masses.

## 1 Introduction

With the advances in the development of neuroimaging tools, neuroscientists have been able to study the human brain anatomy via the construction of 3D surface models, delineating white matter and grey matter structures from magnetic resonance imaging (MRI) scans (Dale et al., 1999;DeKraker et al., 2023;Fischl, 2012). At present many aspiring researchers, lacking in resources, are not able to carry out such research due to various ‘barriers to entry’. First, the acquisition of such MRI data is relatively costly to acquire. Second, the computational resources and equipment to preprocess the raw MRI can also be costly. Third, specialized software and/or operating systems (i.e., Linux-based command line environment [CLE], Matlab, Python) are used for the preprocessing (in themselves or through containers or virtual machines); in most instances, this would require some technical expertise to configure and run the preprocessing pipelines (Fischl, 2012;MacDonald et al., 2000). Fourth, the analysis of such preprocessed data also requires technical expertise and access to computational resources, due to the need to store and convert the preprocessed data into an analysable format and to use specialised software packages that run only in certain operating environments.

With the recent trend in data sharing among neuroimaging researchers via platforms like*OpenNeuro*, as well as the public releases of large-sampled databases from major research consortiums, such as the ABCD and HCP-related studies, aspiring researchers can have a diverse selection of “free” data to work with. In some cases, the preprocessed derivatives are readily available as well. Thus, the first three barriers to entry can be effectively overcome in these, and will not be addressed presently; however, the fourth remains. In particular, FreeSurfer, a software package widely used to preprocess and analyse structural images (Fischl, 2012), can be cumbersome to configure and operate at the post-processing and analysis stages, after the preprocessed data have been obtained. FreeSurfer works in Linux and Mac OS X operating systems but requires dockers or virtual machines to be used on Windows, which adds a layer of inconvenience for the user. The installation and setup of these dockers and virtual machine may require some technical expertise. Moreover, FreeSurfer’s processing analysis pipeline works with a “subject directory” structure where data are stored at each processing step and computation for all subjects, such as surfaces for each hemisphere, brain volumes, and segmentations. These data involve specialized format that restrict statistical analyses to FreeSurfer’s own tools or require conversions into more flexible formats (Schäfer & Andrew, 2022). Furthermore, statistical analyses are typically run by reading from the subject directory environment and, as such, depend on this directory being readily accessible at the time of the analysis. This becomes an obstacle when users do not have convenient or direct access to such subject directory environment.

Additionally, given that tens of gigabytes of disk space are required to store the preprocessed data, such data are often not portable and usually stored on a server or workstation. This means that analyses must be carried out on the server or workstation which presents major inconveniences and difficulties to many. For instance, access to such servers or workstations is not typically available to the masses (i.e., undergraduate psychology/neuroscience students). Furthermore, even if access is granted, one may only be able to access these servers or workstations on the campus network or via a slow and unreliable Virtual Private Network connection, making it difficult for one to work remotely.

**Fig. 1.**
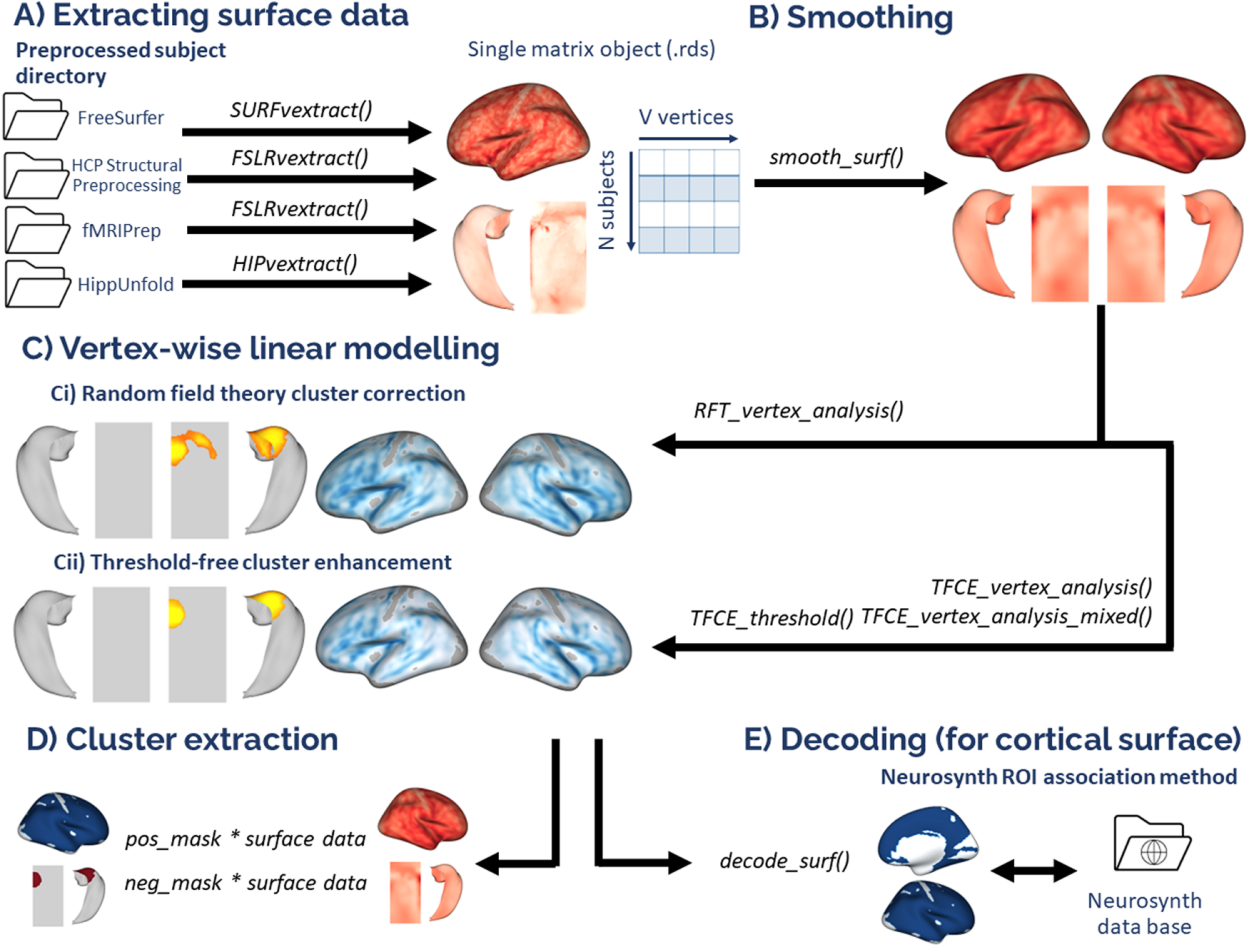
Summary of the VertexWiseR analysis workflow. (A) Extracting the surface data from subjects’ preprocessed output directories. The R functions listed above each arrow work specifically for the output directories of different preprocessing pipelines; these functions output a .rds file containing the surface data from all subjects in the preprocessed output directories within a matrix with the dimensions of N (subjects) x V (vertices). (B) Surface vertex-wise values are smoothed at a user-selected full width at half maximum value (FWHM). (C) Vertex-wise linear models can be analysed with two types of cluster correction methods: random field theory (RFT)-based and threshold-free cluster enhancement (TFCE)-based cluster corrections. (D) Positive and negative cluster masks are outputted from these analyses and the summary vertex-wise values (mean or sum) can be extracted in R by performing a matrix multiplication between the mask and surface data. (E) Using the Neurosynth database (github.com/neurosynth), meta-analytic decoding can be carried out on the clusters to identify keywords that are associated with the clusters.

To these ends, we developed VertexWiseR, which runs on about any computer that runs R, without requiring dockers or virtual machines, and moves the analyses out of the server or workstation. This package does so by making it easy for one to extract the vertex-wise data of various surface templates from the preprocessed subject directories into one single highly compressed file and enabling the user to run analyses solely from that file. As one can imagine, this would be highly appropriate and conducive in classroom settings. VertexWiseR is a user-friendly package working with the R programming language (R Core Team, 2023) that reduces anatomical whole-brain and hippocampal surface data to compact and portable data files and uses the files to compute vertex-wise statistical linear models independently from the base dataset (Fig. 1). While the preprocessed FreeSurfer surface files for all subjects can be summarized and concatenated into a single and portable .mgh file without the need for direct access to the preprocessed subjects directories, no such comparable methods exist for hippocampal and fsLR32k surfaces. VertexWiseR makes it possible to summarize the data from the three supported surface templates into highly compressed .rds files for ease of dissemination and analyses.

The package is the first R package to our knowledge to build analyses functions around compact data separate from subject directories, which are possible in*BrainStat*for Python and Matlab (Larivière et al., 2023). Given R is a language relatively more specific and accessible for statistical analyses (Sarmento & Costa, 2017) as well as popular in statistics courses (behind GUI-based SPSS) (Davidson et al., 2019), R is likely to attract students or researchers interested in applying statistics on neuroimaging data.

In addition, VertexWiseR currently handles cortical surface preprocessed with FreeSurfer as well as cortical surface in the fsLR32k space used in the Human Connectome Project (HCP) (Van Essen et al., 2013) and fMRIprep preprocessing (Esteban et al., 2019) pipelines. On top of adapting conventional surface analysis functions to the R programming language environment, VertexWiseR also handles hippocampal surface data preprocessed with the recently developed HippUnfold pipeline (DeKraker et al., 2023).

Other notable highlights of this R package include meta-analytic decoding of the cortical outputs, which lets the user identify the neuropsychological/task-based correlates previously associated in the literature with the same cortical areas, with reference to a database of task-based fMRI and voxel-based morphometric statistical maps. Additionally to conventional vertex-wise general linear models and random field theory (RFT) based cluster corrections (Larivière et al., 2023), the package offers the possibility to run linear models with threshold-free cluster enhancement (TFCE), which is a method developed to avoid the selection of an arbitrary threshold in cluster formation, assigning to a given cluster the sum of “height” (signal intensity) scores of neighboring voxels (here vertices) multiplied by their extent, following theoretically and empirically defined constants (Smith & Nichols, 2009). The package was designed to be user-friendly and to simplify the computation of the above linear models with the R data structure. We present in this paper the workflow and features of VertexWiseR.

To summarize how VertexWiseR’s functionalities expand on other available R packages supporting surface-based neuroimaging, the availability of various VertexWiseR functionalities in these other R packages is listed inTable 1.

**Table 1.** VertexWiseR package functionalities in parallel to existing R packages.

	VertexWiseR	fsbrain ( Schäfer, 2024 )	Freesurferfomats ( Schäfer & Andrew, 2022 )	CiftiTools ( Pham et al., 2022 )	QDECR ( Lamballais & Muetzel, 2021 )	ggseg ( Mowinckel & Vidal-Piñeiro, 2020 )
Supported surface formats						
FreeSurfer	✓	✓	✓	-	✓	-
HippUnfold	✓	-	-	-	-	-
fsLR32k	✓	-	-	✓	-	-
Plotting						
Cortical surface	✓	✓	-	✓	-	-
Atlas-based ROIs	✓	✓	-	-	-	✓
Subcortical ROIs	-	-	-	✓	-	✓
Whole-brain vertex-wise analyses	✓	-	-	-	✓	-

*Note*. ROI = region-of-interest.

## 2 VertexWiseR Installation

Certain functions in VertexWiseR make use of Python functions from various toolboxes (seeTable 2), which can flexibly be called and executed in R using the*reticulate*package v1.37.0 (Ushey et al., 2023). For reticulate to work properly with VertexWiseR, “Miniconda” (>= v.23.10.0), a lightweight version of Python (Anaconda Inc., 2023), needs to be installed. Alternatively, a Conda environment can be activated before running R, or a Python library or virtualenv can be specified for use (set via reticulate’s use_python(), use_virtualenv()). VertexWiseR includes a function (i.e., VWRfirstrun()) that can be run for the user to select and install all the package’s requirements (such as Python/Miniconda, BrainStat, and BrainStat’s fsaverage templates) at once, requiring very minimal technical expertise and user customization/intervention. When VWRfirstrun() is run, the user is prompted for confirmation at each proposed installation (and only has to type “Yes” or “No” in the R terminal), followed by an automated installation process. Note that beyond the installation of dependencies (which VWRfirstrun() assists with), the user is not required to make any further manual intervention or system configuration—all R and python dependencies are automatically installed when VWRfirstrun() is executed.

**Table 2.** Package components and data that are derived from existing toolboxes.

Toolbox	Version	Components	Used in the following VertexWiseR functions
BrainStat ( Larivière et al., 2023 )	v0.4.2	RFT cluster correction (accessed via reticulate) Mixed effects models (accessed via reticulate) Smoothing function (accessed via reticulate) Surface to volume conversion (accessed via reticulate)	RFT_vertex_analysis() RFT_vertex_analysis(), TFCE_vertex_analysis_mixed() smooth_surf() surf_to_vol()
Nilearn ( Nilearn contributors et al., 2024 )	v0.10.4	TFCE-cluster correction (modified and adapted to R)	TFCE_vertex_analysis(), TFCE_vertex_analysis_mixed(),
NiMARE ( Salo et al., 2022 )	v0.2.2	Meta-analytic decoding (accessed via reticulate)	decode_surf_data()
BrainSpace ( Vos de Wael et al., 2020 )	v0.1.10	Generic surface plots (accessed via reticulate)	plot_surf()
HippUnfold ( DeKraker et al., 2023 )	v1.4.1	Hippocampal plots— (modified and accessed via reticulate)	HIPvextract(), RFT_vertex_analysis(), plot_surf()
freesurferformats	v0.1.18	Function to read freesurfer surface maps	SURFvextract()
ciftiTools	v0.15.1	Function to read cifti (fsLR32k surfaces) files	FSLRvextract()
gifti	v0.8.0	Function to read gifti (hippocampal template) files	HIPvextract()

Data		Source	
fsaverage5, fsaverage6 and fsLR32k templates		BrainStat ( Larivière et al., 2023 )	
Region to vertex mappings the Destrieux atlas (fsaverage5)		Nilearn ( Nilearn contributors et al., 2024 )	
Region to vertex mappings for Desikan, Schaefer and Glasser atlases (fsaverage5)		ENIGMA ( Larivière et al., 2021 )	
Region to vertex mappings for the Schaefer and Glasser atlases (fsLR32k)		Netneurotools ( github.com/netneurolab/netneurotools )	
Region to vertex mappings for the Destrieux and Desikan atlases (fsLR32k)		DiedrichsenLab/fs_LR_32 Github repository ( github.com/DiedrichsenLab/fs_LR_32 )	
Hippocampal template and subfields to vertex mappings		HippUnfold ( DeKraker et al., 2023 )	
Meta-analytic database (modified)		Neurosynth ( github.com/neurosynth )	

## 3 Vertex-Wise Data Extraction and Manipulation

Table 3lists the surface templates that are currently supported by VertexWiseR. The FreeSurfer “recon-all” pipeline outputs vertex-wise data adhering to the fsaverage template, including measures of thickness (Fischl & Dale, 2000), depth/height of vertex (*sulc*), curvature, and surface area. The HippUnfold pipeline segments the hippocampi and recreates a 3D surface mesh in a manner similar to cortical surfaces, outputting vertex-wise measures of thickness, curvature, gyrification, and surface area adhering to the CIT168 template (DeKraker et al., 2023).

**Table 3.** Currently supported surfaces.

Currently supported surfaces	Approximate Vertex spacing (mm)	No. of vertices (both hemispheres)
Cortical surface		
fsaverage5	3.5	20,484
fsaverage6	1.4	81,924
fsLR32k	2	64,984
Hippocampus		
CIT168	0.5	14,524

VertexWiseR provides functions to facilitate and automatize the collation of these bilateral vertex-wise data from all subject’s output directories into a single N (number of subjects) x M (number of vertices) matrix, saved as a highly compressed .rds file format using a single command; that is, SURFvextract() for FreeSurfer (making use of its internal mris_preproc algorithm and freesurferformats); and HIPvextract() for HippUnfold. FSLRvextract() is an additional function that does the same for fsLR32k data processed with the HCP structural preprocessing pipeline and the more recent versions of fMRIprep (using the “—cifti-output 91k” flag) (Esteban et al., 2019).

The package also contains a number of functions that help the user manipulate whole-brain surface data from the matrix objects (Table 4). Additionally, given that the vertex-wise data are in the standard matrix format they can also be easily manipulated with any base R matrix manipulation functions. The package does not support functional (timeseries) data but only static data, where there is only one set of data points (vertices) for each subject in a scan session. That being said, as long as the input matrix object’s dimensions are consistent with any of the supported surface templates, it can be analyzed regardless of whether it contains vertex-wise structural or BOLD activity measurements.

**Table 4.** Secondary functions for surface data object manipulation.

Function	Description
smooth_surf()	Smooths surface data, at defined full width at half maximum (FWHM)
surf_to_atlas()	Returns the mean vertex-wise surface data for each parcel of a selected atlas
atlas_to_surf()	Maps average parcellation data from a selected atlas to a surface
fs5_to_fs6()	Remaps vertex-wise surface data in fsaverage5 space to fsaverage6 space
fs6_to_fs5()	Remaps vertex-wise surface data in fsaverage6 space to fsaverage5 space
surf_to_vol()	Converts surface data to volumetric data (.nii file)

## 4 Example Analyses

### 4.1 Overview

In the subsequent sections, we present two examples to illustrate the analyses of cortical and hippocampal thickness, using linear and mixed-effects models, respectively. Two R markdown generated .html are included in the Supplementary Materials for easy copy and pasting of the analysis code.

### 4.2 Linear model of age and cortical thickness, and meta-analytic decoding

#### 4.2.1 Evaluation data

The publicly accessible Spreng and colleagues’ neurocognitive aging neuroimaging dataset (available at OpenNeuro [doi:10.18112/openneuro.ds003592.v1.0.13; v.1.0.13]) was used in this first example (Spreng et al., 2022). The full dataset consisted of 301 participants from two sites; for the purpose of this demonstration, we have included cortical thickness (CT) and phenotypical data of participants from one of the sites (site 1; Cornell Magnetic Resonance Imaging Facility in New York, USA). Their T1 structural volumes were acquired with a 3T GE Discovery MR750 scanner (32-channel head coil). The MRI protocol was as follows: TR = 2530 ms; TE = 3.4 ms; 7° flip angle; 1 mm isotropic voxels, 176 slices, and 5 minutes and 25 s. The T1-weighted images were preprocessed with the complete recon-all pipeline in FreeSurfer v.7.2.0 (Fischl, 2012). This included sample was composed of 238 healthy adults, with younger participants (N = 154; Mean age = 22.29y ± 3.12; 56% female) and older participants (N = 84; mean age = 67.54y ± 5.69; 56% female). A .csv sheet containing demographics data (from the participant.tsv sheet) and accompanying behavioral data (fromosf.io,doi:10.17605/OSF.IO/YHZXE) was created in R and included in the package’s inst/demo_data/ folder.

#### 4.2.2 Preparing the surface data

**Fig. 2.**
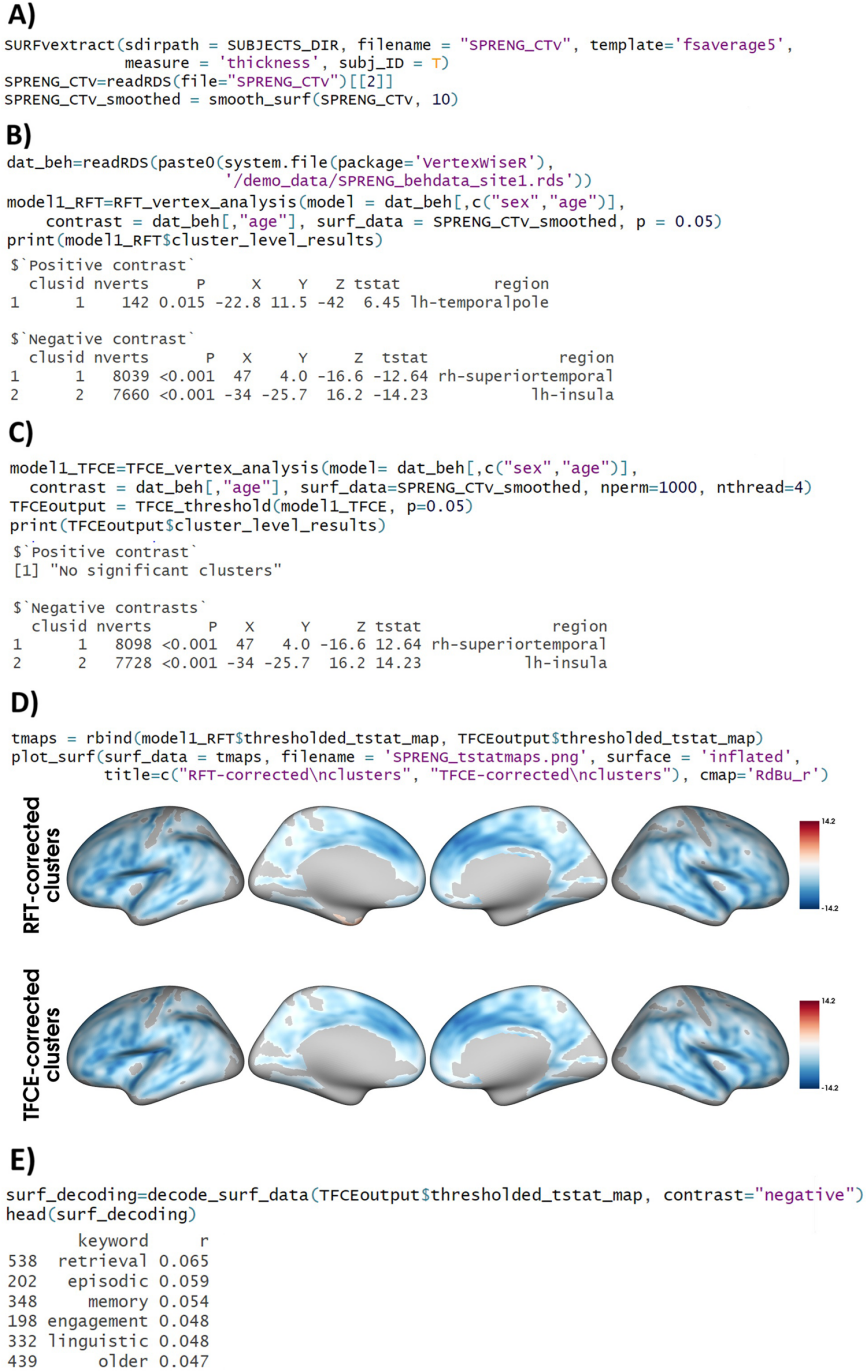
Code and output for example 1. Note. (A) Extracting surface data from the FreeSurfer subjects directory and creating a single .rds file. The surface data are then smoothed at 10 FWHM. (B) Vertex-wise linear model with random field theory (RFT)-based cluster correction, testing for the effect of age on cortical thickness, controlling for sex. (C) Vertex-wise linear model with threshold-free cluster enhancement (TFCE)-based cluster correction, with 1000 permutations, testing for the effect of age on cortical thickness, controlling for sex, using 4 CPU threads. (D) Plotting the thresholded t-value maps obtained from both the RFT-based and TFCE-based linear models on an inflated fsaverage5 surface template. (E) Meta-analytic decoding of the significant negative clusters from a neuroimaging database. RFT = Random field theory. TFCE = Threshold-free cluster enhancement. In the $cluster_level_results output of (B) and (C), the “X”, “Y”, and “Z” values represent the MNI coordinates of the cluster peak; the “tstat” (t-statistic) reported belongs to this peak and “region” identifies the region which this peak belongs to; and “P” refers to the cluster-wise corrected p-value.

In this demonstration, CT in each subject was extracted from the dataset. The surface data was resampled to fsaverage5 and stored as a matrix R object in the SRENG_CTv.rds file using the SURFvextract() function (Fig. 2A)*.*The output SRENG_CTv.rds contained a list of subject IDs (option given with the argument subj_ID), and a matrix made of N rows per participant (ordered as in the FreeSurfer subject directory) and 20484 columns (N vertices in the fsaverage5, from left to right hemisphere). Each vertex had a CT value in the matrix. The surface data were smoothed with a 10 mm FWHM kernel for the SPRENG_CTv object with the smooth_surf() function (Fig. 2A).

#### 4.2.3 Vertex-wise linear modelling

The effect of age on cortical thickness was investigated in a whole-brain vertex-wise model, controlling for sex, with the code described inFigure 2B. The*model*argument corresponded to a data frame object containing the Spreng dataset demographics information (sex and age).*Contrast*was the variable of interest (which was here part of the same data frame), and surf_data was the matrix object outputted by SURFvextract() and subsequently smoothed. This model was then fitted to a standard linear regression model (via BrainStat’s brainstat.stats.SLM function) using the RFT_vertex_analysis() command. The significant clusters predicted by the contrast variable were thresholded using BrainStat’s RFT approach (Larivière et al., 2023;Worsley et al., 1999) at a user-defined threshold*p*(default 0.05; used in the current example).

The output of the analysis was a list object containing the tabulated results of the significant clusters where age had a positive or negative effect on CT (Fig. 2B), and additional statistical maps as shown inTable 5. These maps can be easily submitted to the plot_surf() function for plotting.

**Table 5.** Statistical maps generated in the analysis.

Maps	Description
thresholded_stat_map	Cluster-thresholded t-statistical map
pos_mask	Binarized map of all significant positive clusters
neg_mask	Binarized map of all significant negative clusters
pos_clusterIDmap	Map of all significant positive clusters, labelled according to their cluster membership
neg_clusterIDmap	Map of all significant negative clusters, labelled according to their cluster membership

#### 4.2.4 Threshold-free cluster enhancement

The TFCE_vertex_analysis() function allows the user to analyse a model and threshold clusters using the TFCE approach (Smith & Nichols, 2009). TFCE values for each cluster are estimated using the parameters of extent (E)=1 and height (H)=2 as recommended for two-dimensional data (Smith et al., 2006;Smith & Nichols, 2009). These TFCE values are thresholded against a null distribution of TFCE values generated by permuting the subjects’ data (i.e., shuffling the rows in the surf_data matrix). The number of permutations is set at 100 by default and can be specified by the user. Unlike the RFT approach, the cluster p-value threshold is not specified at the outset; instead, a separate function TFCE_threshold() is needed to threshold the TFCE-corrected results. This is designed such that one can adjust this*p*-value threshold without repeating the lengthy TFCE estimation procedure.

The same linear model analysis above was fitted with TFCE_vertex_analysis(), and the number of permutations for estimating the null distribution was set at 1000 (Fig. 2C)*.*To identify significant clusters at the desired threshold (set at*p*< 0.05 for this example) from the permutations, we used the TFCE_threshold() function. The format of the output list object was similar to that of the regular vertex-wise analysis. In this analysis, only the negative clusters were significant (Fig. 2C).

Figure 2Ddepicts the output of plot_surf(), used to produce a figure from the t-values maps of both models (the RFT cluster-corrected model and the TFCE-corrected model together). The rbind function allowed multiple maps to be concatenated in a single plot. Each map can be attributed its own title and color map as shown inFigure 2Dcode.

#### 4.2.5 Meta-analytic decoding of the vertex-wise data

We implemented a meta-analytic decoder to help users to make sense of the obtained clusters and contextualize the findings in the wider task-based fMRI literature. VertexWiseR uses the Python-integrated package Neuroimaging Meta-Analysis Research Environment (NiMARE) (Salo et al., 2022) to read into the Neurosynth database (imported fromhttps://github.com/neurosynth/neurosynthdata version 0.7, July 2018) and use the database to decode a given ROI mask. The Neurosynth database contains activation maps from 14,371 task-based functional magnetic resonance imaging (fMRI) studies. Each of these activation maps is labeled with keywords pertaining to the task or stimulus that elicits the activation. The decode_surf_data() function uses the ROI-association approach to calculate label-wise correlations associated with the given ROI mask. The output of this function is a data.frame object containing the keywords and their label-wise correlation values, ordered according to the magnitudes of the correlation coefficient.

To minimize computation time and irrelevant results, keywords were removed from the package database if they were: the name of an anatomical brain region, protocol-specific descriptive words that are not meaningful out of context (e.g., “studied”, “clips”, “engaged”), and broad categories that do not specifically point to a content (“disorder”, “psychological”, “cognitive”). This function currently only works with statistical maps in fsaverage5 space.

The significant negative clusters from the previously reported whole-brain TFCE analysis were “decoded” for vertices within negative clusters as shown inFigure 2E. decode_surf_data() returns a data.frame object listing all keywords from the database and the latter’s correlation (*r*) with the surface data (Fig. 2E).

### 4.3 Mixed-effect model of intervention-related changes on hippocampal thickness

#### 4.3.1 Evaluation data

In this second example, the whole hippocampal vertex-wise thickness data from an intervention study (Fink et al., 2021) (available at OpenNeurohttps://doi.org/10.18112/openneuro.ds003799.v2.0.0; v. 2.0.0) was analyzed using a mixed-effects model. This dataset was also included in the R package as another demonstration dataset. It was composed of 48 young adults who had MRI scans at three time-points of a two-week running intervention, with one group who received the intervention between t1 and t2 (N = 21; mean age = 23.24 y ± 3.18; 57% female), and a second group between t2 and t3 (N = 27; mean age = 22.78 y ± 2.58; 63% female). A .csv sheet containing demographics and behavioral data was created in R based on the dataset’s participant.tsv and CES-D.tsv files and included in the package’s inst/demo_data/ folder for demonstration. For the present analysis, groups were coded -1 (group 1) and 1 (group 2). Each participant had a 3T T1-weighted MRI scan obtained with a 3T Tim-Dot Magnetom Skyra system with syngo MR E11 (Siemens Medical Systems, Erlangen, Germany), with the following protocol: TR= 2200 ms; TE = 2.18 ms, 8° flip angle; 0.88 mm isotropic voxels, 224 slices, and 5 minutes 51 s. The T1-weighted images were preprocessed with the default settings of the HippUnfold v.1.4.1 pipeline.

#### 4.3.2 Preparing the surface data

**Fig. 3.**
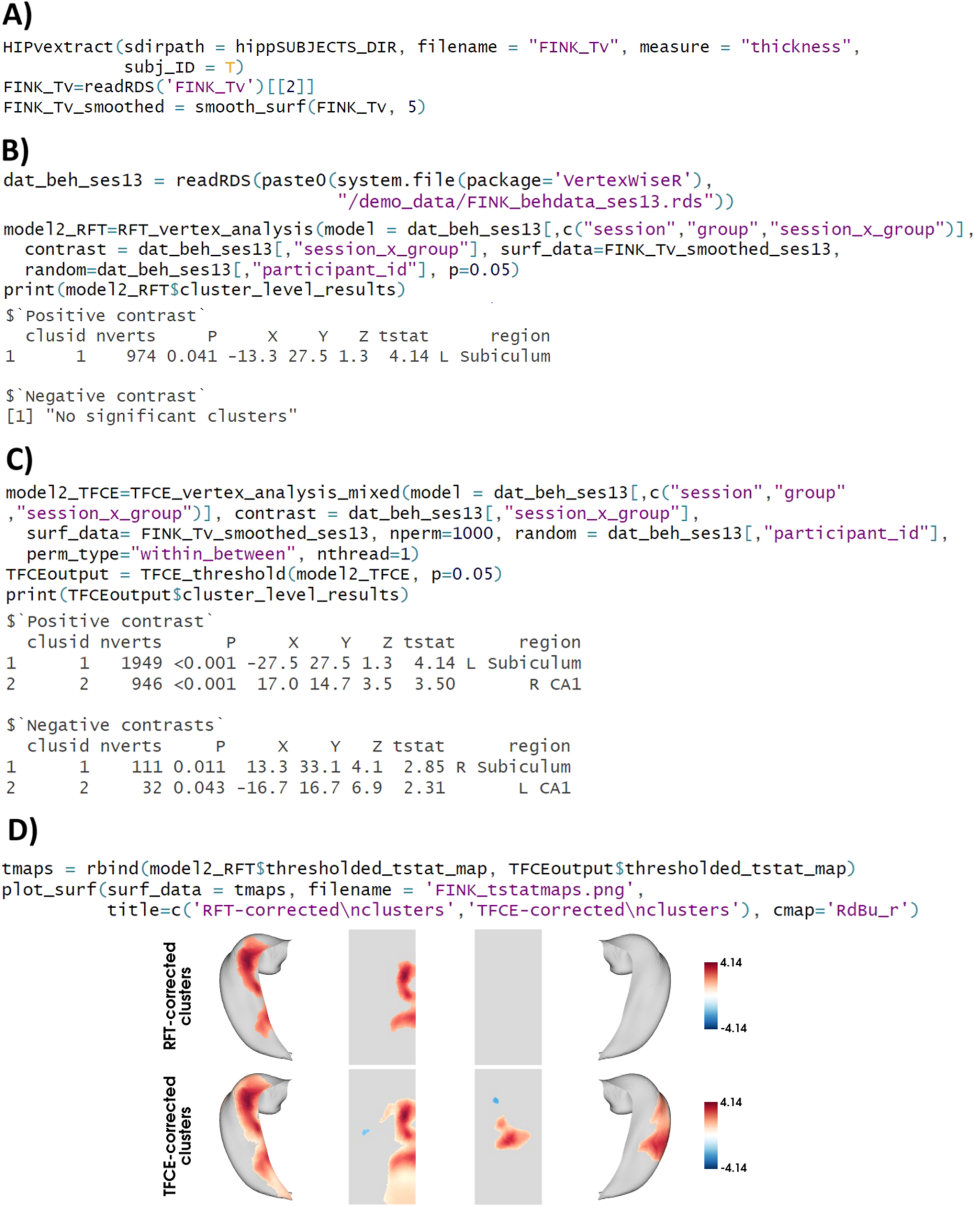
Code and output for example 2. Note. (A) Extracting surface data from the HippUnfold subjects directory and creating a single .rds file. The surface data are then smoothed at 5 FWHM. (B) Vertex-wise linear model with random field theory (RFT)-based cluster correction, testing for the effect of group, time, and their interaction, with subject ID as a random variable. (C) Vertex-wise linear model with threshold-free cluster enhancement (TFCE)-based cluster correction, with 1000 permutations, testing for the effect of group, time, and their interaction, with subject ID as a random variable, using 1 CPU thread. (D) Plotting the thresholded t-value maps obtained from both the RFT-based and TFCE-based linear models on a CIT168 surface template. RFT = Random field theory. TFCE = Threshold-free cluster enhancement. In the $cluster_level_results output of (B) and( C), the “X”, “Y” and “Z” values represent the MNI coordinates of the cluster peak; the “tstat” (t-statistic) reported belongs to this peak and “region” identifies the region which this peak belongs to; and “P” refers to the cluster-wise corrected p-value.

Similarly, bilateral hippocampal thickness data (in the*HippUnfold*subjects directory) were extracted as a matrix and stored in an R list object (FINK_Tv.rds) with the HIPvextract() function (Fig. 3A). The output FINK_Tv likewise contained a list of subject IDs (option given with the argument subj_ID) and a matrix made of N rows per participant (ordered as in the HippUnfold subject directory) and 14,524 columns for vertices, corresponding to the left then right hippocampus. Each vertex had a thickness value in the matrix. The surface data were smoothed with a 5 mm FWHM kernel for the hippocampal FINK_Tv surface (Fig. 3A).

**Fig. 4.**
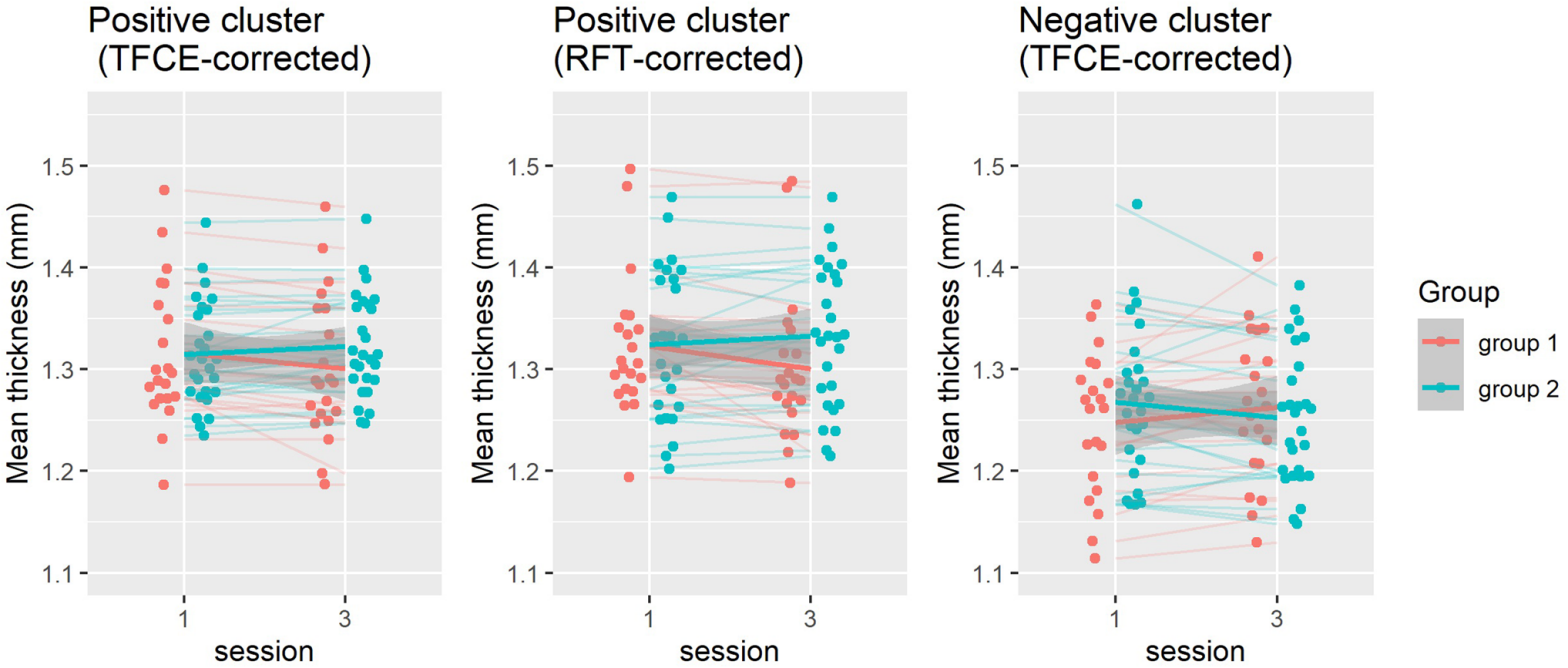
Hippocampal clusters where an interaction effect of time and group was found using random field theory and threshold-free cluster enhancement

#### 4.3.3 Standard hippocampal vertex-wise mixed linear model

Thickness was investigated in a vertex-wise model testing for the interaction of time (t1 and t3) and group (1 and 2) on hippocampal thickness (Fig. 3B). The*model*argument corresponded to a data frame object containing the demographics information (session (time point); group; group*session interaction variable). The interaction variable was set as the contrast, the random variable was participant ID, and surf_data was the matrix object outputted by HIPvextract() including only ses-1 and ses-3 surface outputs. The significant clusters predicted by the contrast variable were thresholded using the same BrainStat’s RFT approach (Larivière et al., 2023;Worsley et al., 1999) at a user-defined threshold*p*(default 0.05; used in the current example). The results of the significant clusters are shown inFigure 3B.

#### 4.3.4 Hippocampal vertex-wise mixed linear model with threshold-free cluster enhancement

The same model as in the previous section was re-analyzed using TFCE with 1000 permutations. With the “perm_type” option, the user can select the type of permutation to apply to the data: shuffle all rows (default), shuffle rows within subjects rows, between subjects (without altering the order within subjects), or both within and between subjects. Here, the data were shuffled within and between subjects. Five significant clusters were observed (Fig. 3C). Like in the whole-brain models, the plot_surf() function can be used on the hippocampal surface (Fig. 3D).

In this example, TFCE appears to be the more liberal thresholding approach. The within and between-subject permutations made it easier for the analysis to detect a significant within-between-subject interaction effect; no significant clusters were detected if the default row-based permutation was carried out.Figure 4displays the thickness of the positive and negative clusters in relation to the group and session variables, in RFT and TFCE models, demonstrating a steeper curve toward group 2. The plots were produced using the negative and positive masks and the ggplot2 package v.3.5.1 (Whickam, 2016).

As an additional validation of these results, these significant clusters were extracted as regions-of-interests and fitted in a linear mixed-effects model using another R package—lmerTest (Kuznetsova et al., 2017). With the different masks outputted by each analysis function (RFT_vertex_analysis(), TFCE_vertex_analysis_mixed(), TFCE_vertex_analysis()), users can extract the clusters defined by these masks as ROI values, by simply carrying out a matrix multiplication between the $pos_mask or $neg_mask object (which are binary masks) and the surface data. For example:

cluster _ T=FINKv  % * %   TFCEoutput$pos_mask

“%*%” is the operator to execute matrix multiplication. Therefore, this line will add up, for each subject, all the thickness values in the included vertices (coded as 1 s in the mask), giving a total thickness measure for each subject. This total thickness measure might be somewhat difficult to interpret or relate to, hence we can further divide these values by the number of included vertices (arithmetic sum of the mask) to obtain the average (per vertex) thickness values.

Using the lmerTest package (Kuznetsova et al., 2017) to run the linear mixed models separately testing cluster-wise thickness data, the interaction variable was found to show a significant effect on hippocampal thickness (*β*= 0.0076;*t*= 4.84;*p*= 0.00002); with more sensitivity in the TFCE model (*β*= 0.0056;*t*= 4.99;*p*< 0.00001). The code used to obtain this data is attached in the Supplementary Materials S1.

### 4.4 Completion times for TFCE analyses

Both example analysis pipelines previously described were carried out on a Windows 11 system using an Intel(R) Core(TM) i7-8565U CPU @ 1.8GHz (released in 2018), with 16 GB RAM; and on an AMD Ryzen 7 5700X @ 3.4GHz (released in 2022), with 16 GB RAM.Table 6lists the time taken to complete each statistical analysis with different numbers of Central Processing Unit (CPU) threads (specified with the*nthread*argument in the TFCE functions). According to these completion times, it appears that the TFCE analyses do not run as efficiently via parallel processing on older CPUs, especially in the second example where the imported Python function was run in parallel within the R environment for the mixed TFCE model.

**Table 6.** Example threshold-free cluster enhanced-based analyses durations depending on the number of allocated threads.

CPU system	nthread = 1	nthread = 2	nthread = 4
Example 1			
Intel(R) Core(TM) i7-8565U CPU @ 1.8GHz	75.3 minutes	51.2 minutes	30.1 minutes
AMD Ryzen 7 5700X @ 3.4GHz	64.2 minutes	32.2 minutes	16.5 minutes
Example 2			
Intel(R) Core(TM) i7-8565U CPU @ 1.8GHz	100.1 minutes	148.3 minutes	266.1 minutes
AMD Ryzen 7 5700X @ 3.4GHz	62.0 minutes	31.0 minutes	17.4 minutes

*Note*. CPU = central processing unit.

## 5 Discussion

VertexWiseR provides tools to run vertex-wise statistical models in R, with both whole-brain and hippocampal surfaces. Its specificity is to reduce cohort surface data to easy-to-manipulate objects which can be used later for statistical computation independently from access to the base cohort sample. While offering the conventional vertex-wise linear models and cluster correction, the package also includes threshold-free cluster enhancement, which was shown here to exclude potential cluster-wise false positives. VertexWiseR aims to facilitate the application of statistical models for neuroscience researchers investigating surface-based structural features of the cortical and hippocampal surface.

As shown inTable 7, VertexWiseR borrows some of the best and most useful functions from different toolboxes, and builds them into a single R package. While it is possible to manually combine these functions from the different toolboxes to accomplish a set of tasks (e.g., reading FreeSurfer output directories, fitting a mixed-effect model, estimating TFCE statistics and meta-analytic decoding), this would be somewhat inconvenient and cumbersome. One would need to “code-switch” between different programming languages, and also to reformat the output from one task in a toolbox so that it can be entered as an input for a subsequent task in another toolbox. To this end, our package connects each of these tasks seamlessly, such that the output of an earlier task can be easily and directly entered as an input in the next task.

**Table 7.** Comparing between various vertex-based analysis toolboxes/software.

	VertexWiseR	QDECR ( Lamballais & Muetzel, 2021 )	BrainStat ( Larivière et al., 2023 )	FreeSurfer ( Fischl, 2012 )	CAT12 ( Gaser et al., 2024 )	TFCE_mediation ( Lett et al., 2017 )
Operating systems (outside of dockers/virtual machines)	Windows, Linux, MacOS	Linux, MacOS	Windows, Linux, MacOS	Linux, MacOS	Windows, Linux, MacOS	Linux, MacOS
Environment	R	R	Python/MATLAB	CLE	MATLAB GUI	CLE
Preprocess raw data	No	No	No	Yes	Yes	No
Reads surface data from preprocessed output directories	Yes (FreeSurfer, HCP pipeline, HippUnfold, fMRIprep)	Yes (FreeSurfer)	No	Yes (FreeSurfer)	Yes (CAT12)	Yes (FreeSurfer, CIVET)
Concatenates all subjects’ data into a single portable file	Yes (.rds format)	No	No	Yes (.mgh format)	No	Yes (.mgh format)
Surface supported	fsaverage5, fsaverage6,fsLR32k, CIT168	All fsaverage	All fsaverage, fsLR32k, CIVET	All fsaverage	fsLR32k	All fsaverage, CIVET
Cluster thresholding procedure	RFT, TFCE	MCS	RFT	MCS	TFCE, FWE, FDR	TFCE
Supported statistical models	Linear, Mixed-effects	Linear	Linear, Mixed-effects	Linear, Mixed-effects	Linear, Flexible factorial	Linear, Mediation
Extract atlas-based ROIs	Yes	No	No	Yes	Yes	No
Meta-analytic decoding	Yes	No	Yes	No	No	No

*Note*. CLE = command line environment; GUI = graphical user interface; RFT = random field theory; FDR = false discovery rate; TFCE = threshold-free cluster enhancement; MCS = Monte Carlo simulation; FWE = family wise error.

This is not to discount the fact that our package includes some unique functions of our own. For instance, VertexWiseR is the only toolbox that is capable of 1) reading data from HCP structural processing, HippUnfold and fMRIprep preprocessed directories, 2) native support for the CIT168 hippocampal surface, and 3) concatenating all subjects’ data into a highly compressed .rds file—which is roughly two-third the total file size of .mgh files (both hemispheres) from the same set of subjects. Thus, VertexWiseR is arguably one of the most versatile tool for analyzing vertex-wise data to date.

To demonstrate the use of the R package, we have carried out two example analyses using publicly accessible datasets. The first example reinforced the widespread effect of age on cortical thinning in older adults and how thickness can be a sensitive measure of neurodegeneration in the typical aging population (Fjell et al., 2014;Frangou et al., 2022;van Velsen et al., 2013). In the second example, we were also able to reproduce the results of Fink and colleagues, pertaining to intervention-related gains in hippocampal volume (Fink et al., 2021), with our analysis of hippocampal thickness at the vertex-wise level. This demonstrates the complementary information surface-based measures that can be obtained with VertexWiseR. These example analyses highlighted the ease at which one can run analyses to identify significant clusters as a function of individual differences, and longitudinal/intervention-related changes. These examples should cover most analysis scenarios in relation to brain morphological changes.

In this R package, we have provided two different means of thresholding the clusters. TFCE clustering in functional MRI data was found to be more sensitive to true voxel-wise effects compared to RFT clustering, especially for medium and lower effect sizes (Noble et al., 2020;Smith & Nichols, 2009). In a previous study, TFCE had a higher spatial bias towards detecting findings in visual cortical areas extending to the posterior cingulate cortex, as opposed to CSF-adjacent and frontal areas (Noble et al., 2020). Thus, there may be a trade-off between reducing bias toward specific areas and detecting effects of medium and below size, that VertexWiseR lets the users choose, and may explain the present “discrepancy” between RFT-corrected and TFCE-corrected hippocampal clusters.

Currently, most neuroimaging analysis toolboxes are Python- or Matlab-based. Thus, our R package may seem somewhat unconventional in this regard. Being specific to R could limit its convenience to users with base-level experience in the R programming language. Nevertheless, R is widely used in psychology research; it is increasingly being taught to psychology and medical students and has been positively received by them (Counsell & Cribbie, 2020;da Silva & Moura, 2020). Given the relevance of brain imaging in the psychology and medicine disciplines, our R package is aptly positioned to serve researchers and students in these disciplines. The ease of use demonstrated in the R package also makes it an ideal first exposure to neuroimaging analyses for these students. It is hoped that our R package will inspire the development of future R packages to expand the use of R in the neuroimaging field.

Limitations to the package include the current limitation to specific surface spaces (FreeSurfer’s fsaverage template space and HippUnfold’s template space). VertexWiseR in its current form is not compatible with other surface reconstruction software (Diers et al., 2023;Gaser et al., 2024;MacDonald et al., 2000). Nevertheless, additional surface templates can be easily incorporated into future updates of the R package if they are widely requested. At present, the fsaverage template (327,684 vertices) is not supported because the analyses of data in the fsaverage space, especially those involving TFCE, will take up a lot more computational resources (i.e., computation time, storage space, memory) which would not be feasible for the typical student’s laptop.

## Ethics

The Spreng and colleagues dataset used for demonstration was acquired in compliance with the Institutional Review Board at Cornell University and the Research Ethics Board at York University, including the participant’s written informed consent (Spreng et al., 2022). Likewise, data acquired as part of the Fink and colleagues dataset used for present demonstration included written informed consent from participants and received approval by an authorized ethics committee (Fink et al., 2021).

## Supplementary Material

Supplementary Material 1

## Data Availability

The Spreng and colleagues’ neurocognitive aging neuroimaging dataset is available on OpenNeuro: doi:10.18112/openneuro.ds003592.v1.0.13 The Fink and colleagues’ whole hippocampal vertex-wise thickness data from an intervention study is available on OpenNeuro:https://doi.org/10.18112/openneuro.ds003799.v2.0.0 The code produced to develop VertexWiseR is made publicly available on its git repository athttps://github.com/CogBrainHealthLab/VertexWiseR
