# Supplementary material for: VertexWiseR: A package for simplified vertex-wise analyses of whole-brain and hippocampal surfaces in R
Source: Imaging Neurosci (Camb). 2024 Nov 14;2:imag-2-00372. doi: 10.1162/imag_a_00372 (PMC12330379; doi:10.1162/imag_a_00372)
Supplement: Supplementary Material 1 [file imag_a_00372-supp1.zip › imag_a_00372-supp1.html]

Example analyses with VertexWiseR - Example 1


# Example analyses with VertexWiseR - Example 1

#### Charly Billaud, Junhong Yu

#### 2024-08-12

## Installing VertexWiseR and checking for requirements

The following code installs the package and makes sure all
requirements, prompting the user to install dependencies, in order to
allow analyses to work.

```
install.packages("VertexWiseR")
```

```
library(VertexWiseR)
```

VWRfirstrun() checks all system requiremements for specific
functions, and gives the opportunity to download and install each of
them.

```
VWRfirstrun()
```

## Example analysis 1: linear model of age and cortical thickness, and meta-analytic decoding

The first stage of the analysis assumes that a preprocessed
Freesurfer subjects directory is present. However, this code makes use
of an already-extracted cortical thickness (CT) dataset made available
on the VertexWiseR git repository.

The following commented out code is the script which was used to
produce this demo data with the SPRENG
dataset (Spreng et al. 2022):

```
#SURFvextract(sdirpath = SUBJECTS_DIR, filename = "SPRENG_CTv", template='fsaverage5', measure = 'thickness', subj_ID = T)
```

The surface can be loaded straight from the online repository and
then smoothed :

```
SPRENG_CTv = readRDS(file = url("https://github.com/CogBrainHealthLab/VertexWiseR/blob/main/inst/demo_data/SPRENG_CTv_site1.rds?raw=TRUE"))

SPRENG_CTv_smoothed = smooth_surf(SPRENG_CTv, 10)
```

The SPRENG behavioural data (for participants in site 1,
SPRENG\_behdata\_site1.csv) is accessible from the VertexWiseR package
internal data:

```
dat_beh=readRDS(system.file(package='VertexWiseR', '/demo_data/SPRENG_behdata_site1.rds'))
```

To run the vertex-wise model analysis with random field theory-based
cluster correction, testing for the effect of age, controlling for sex,
on CT:

```
model1_RFT=RFT_vertex_analysis(model = dat_beh[,c("sex","age")], 
  contrast = dat_beh[,"age"], surf_data = SPRENG_CTv_smoothed, p = 0.05)
```

```
model1_RFT$cluster_level_results
```

```
## $`Positive contrast`
##   clusid nverts     P     X    Y   Z tstat          region
## 1      1    142 0.015 -22.8 11.5 -42  6.45 lh-temporalpole
## 
## $`Negative contrast`
##   clusid nverts      P   X     Y     Z  tstat              region
## 1      1   8039 <0.001  47   4.0 -16.6 -12.64 rh-superiortemporal
## 2      2   7660 <0.001 -34 -25.7  16.2 -14.23           lh-insula
```

To run the vertex-wise model analysis with threshold-free cluster
enhancement-based cluster correction, testing for the effect of age,
controlling for sex, on CT; with 1000 permutations:

```
model1_TFCE=TFCE_vertex_analysis(model= dat_beh[,c("sex","age")], 
                                 contrast = dat_beh[,"age"],
                                 surf_data=SPRENG_CTv_smoothed,
                                 nperm=1000, 
                                 nthread=4) 

TFCEoutput = TFCE_threshold(model1_TFCE, p=0.05)
```

```
TFCEoutput$cluster_level_results
```

```
## $`Positive contrast`
## [1] "No significant clusters"
## 
## $`Negative contrasts`
##   clusid nverts      P   X     Y     Z tstat              region
## 1      1   8098 <0.001  47   4.0 -16.6 12.64 rh-superiortemporal
## 2      2   7728 <0.001 -34 -25.7  16.2 14.23           lh-insula
```

To plot the results of both models on an inflated fsaverage5
surface:

```
tmaps = rbind(model1_RFT$thresholded_tstat_map, TFCEoutput$thresholded_tstat_map)
plot_surf(surf_data = tmaps, 
          filename ='SPRENG_tstatmaps.png', 
          surface = 'inflated', 
          title=c("RFT-corrected\nclusters", "TFCE-corrected\nclusters"),
          cmap='RdBu_r',
          show.plot.window=FALSE)
```

To run meta-analytic decoding of the significant negative clusters
(the neurosynth dataset needs to be installed as VWRfirstrun()
allows):

```
surf_decoding=decode_surf_data(TFCEoutput$thresholded_tstat_map, contrast="negative")
```

```
head(surf_decoding)
```

```
##        keyword     r
## 538  retrieval 0.065
## 202   episodic 0.059
## 348     memory 0.054
## 198 engagement 0.048
## 332 linguistic 0.048
## 439      older 0.047
```

## References:

Spreng, R. Nathan, Roni Setton, Udi Alter, Benjamin N. Cassidy, Bri
Darboh, Elizabeth DuPre, Karin Kantarovich, et al. 2022.
“Neurocognitive Aging Data Release with Behavioral, Structural and
Multi-Echo Functional MRI Measures.” *Scientific
Data* 9 (1): 119. https://doi.org/10.1038/s41597-022-01231-7.
