# Supplementary material for: VertexWiseR: A package for simplified vertex-wise analyses of whole-brain and hippocampal surfaces in R
Source: Imaging Neurosci (Camb). 2024 Nov 14;2:imag-2-00372. doi: 10.1162/imag_a_00372 (PMC12330379; doi:10.1162/imag_a_00372)
Supplement: Supplementary Material 1 [file imag_a_00372-supp1.zip › imag_a_00372-supp2.html]

Example analyses with VertexWiseR - Example 2


# Example analyses with VertexWiseR - Example 2

#### Charly Billaud, Junhong Yu

#### 2024-08-12

## Example 2: Mixed effect model of intervention-related changes on hippocampal thickness

The first stage of the analysis assumes that a preprocessed
Hippunfold subjects directory is present. Likewise, this code makes use
of an already-extracted hippocampal thickness dataset made available on
the VertexWiseR git repository.

The following code is the script which was used to produce this demo
data with the Fink
dataset (Fink et al. 2021):

```
#HIPvextract(sdirpath = hippunfold_SUBJECTS_DIR, filename = "FINK_Tv", measure = "thickness", subj_ID = T)
```

The hippocampal surface can be loaded from the online VertexWiseR git
repository.

To load the thickness matrix:

```
FINK_Tv_ses13 = readRDS(file = url("https://github.com/CogBrainHealthLab/VertexWiseR/blob/main/inst/demo_data/FINK_Tv_ses13.rds?raw=TRUE"))
```

To smooth the surface data:

```
FINK_Tv_smoothed_ses13 = smooth_surf(FINK_Tv_ses13, 5)
```

The FINK behavioural data (FINK\_behdata\_ses13.csv) can be loaded from
the VertexWiseR package internal data. It contains one row per
participant, for scanning sessions 1 and 3.

```
dat_beh_ses13 = readRDS(system.file(package='VertexWiseR', "/demo_data/FINK_behdata_ses13.rds"))
```

Here, we are interested in the interaction between session number
(time) and group. To run the vertex-wise mixed model analysis with
random field theory-based cluster correction, testing for the effect of
session, group, session \* group interaction, on hippocampal thickness,
with subject ID as a random variable:

```
model2_RFT=RFT_vertex_analysis(
  model = dat_beh_ses13[,c("session","group","session_x_group")],
  contrast = dat_beh_ses13[,"session_x_group"],
  surf_data=FINK_Tv_smoothed_ses13,
  random=dat_beh_ses13[,"participant_id"], p=0.05)
```

```
model2_RFT$cluster_level_results
```

```
## $`Positive contrast`
##   clusid nverts     P     X    Y   Z tstat      region
## 1      1    974 0.041 -13.3 27.5 1.3  4.14 L Subiculum
## 
## $`Negative contrast`
## [1] "No significant clusters"
```

To run the vertex-wise mixed model analysis with threshold-free
cluster enhancement-based cluster correction, with 1000 permutations,
testing for the effect of session, group, session \* group interaction,
on hippocampal thickness, with subject ID as a random variable:

```
model2_TFCE=TFCE_vertex_analysis_mixed(
  model = dat_beh_ses13[,c("session","group","session_x_group")], 
  contrast = dat_beh_ses13[,"session_x_group"], 
  surf_data= FINK_Tv_smoothed_ses13, 
  nperm=1000, 
  random = dat_beh_ses13[,"participant_id"], 
  perm_type="within_between", 
  nthread=1) 
TFCEoutput = TFCE_threshold(model2_TFCE, p=0.05)
```

```
TFCEoutput$cluster_level_results
```

```
## $`Positive contrast`
##   clusid nverts      P     X    Y   Z tstat      region
## 1      1   1993 <0.001 -27.5 27.5 1.3  4.14 L Subiculum
## 2      2    972 <0.001  17.0 14.7 3.5  3.50       R CA1
## 
## $`Negative contrasts`
##   clusid nverts     P     X    Y   Z tstat      region
## 1      1    121 0.008  13.3 33.1 4.1  2.85 R Subiculum
## 2      2     68 0.039 -16.7 16.7 6.9  2.31       L CA1
## 3      3     50 0.044 -18.5 18.5 3.8  2.17       L CA1
```

To plot the significant clusters from both models on the CITI168
hippocampal template surface:

```
tmaps = rbind(model2_RFT$thresholded_tstat_map, TFCEoutput$thresholded_tstat_map)
plot_surf(surf_data = tmaps, 
          filename = 'FINK_tstatmaps.png', 
          title=c('RFT-corrected\nclusters','TFCE-corrected\nclusters'), 
          cmap='RdBu_r',
          show.plot.window=FALSE)
```

## Example 2 follow-up: plotting and post-hoc analyses of hippocampal clusters across regression models

The code below was used in R (v.4.3.3) to plot the cluster-wise
values from the RFT and TFCE corrected analyses and validate them with
additional mixed linear models.

We produce a figure displaying the thickness of the positive and
negative hippocampal clusters in relation to the group and session
variables, in RFT and TFCE models, demonstrating a steeper curve toward
group 2:

```
#We divide the cluster values by their sum to get the average thickness per vertex
dat_beh_ses13$clustCTTFCE=(FINK_Tv_smoothed_ses13 %*% TFCEoutput$pos_mask)/sum(TFCEoutput$pos_mask>0)
dat_beh_ses13$clustRFT=(FINK_Tv_smoothed_ses13 %*% model2_RFT$pos_mask)/sum(model2_RFT$pos_mask>0)
dat_beh_ses13$neg.clustCTTFCE=(FINK_Tv_smoothed_ses13 %*% TFCEoutput$neg_mask)/sum(TFCEoutput$neg_mask>0)

library(ggplot2)
library(ggbeeswarm)
library(cowplot)

a=ggplot(data=dat_beh_ses13,aes(y=clustCTTFCE,x=as.factor(session), color=as.factor(group)))+
  geom_quasirandom(dodge.width=0.5)+
  geom_line(aes(group=participant_id), alpha=0.2)+
  geom_smooth(aes(group=group), method="lm")+
  labs(y="Mean thickness (mm)", x="session", color="group")+
  guides(colour = "none")+
  ggtitle("Positive cluster\n (TFCE-corrected)")+
  ylim(1.1, 1.55)
  
b=ggplot(data=dat_beh_ses13,aes(y=clustRFT,x=as.factor(session), color=as.factor(group)))+
  geom_quasirandom(dodge.width=0.5)+
  geom_line(aes(group=participant_id), alpha=0.2)+
  geom_smooth(aes(group=group), method="lm")+
  labs(y="Mean thickness (mm)", x="session", color="group")+
  guides(colour = "none")+
  ggtitle("Positive cluster\n(RFT-corrected)")+ 
  ylim(1.1, 1.55)

c=ggplot(data=dat_beh_ses13,aes(y=neg.clustCTTFCE,x=as.factor(session), color=as.factor(group)))+
  geom_quasirandom(dodge.width=0.5)+
  geom_line(aes(group=participant_id), alpha=0.2)+
  geom_smooth(aes(group=group), method="lm")+
  labs(y="Mean thickness (mm)", x="session", color="group")+
  ggtitle("Negative cluster\n(TFCE-corrected)")+
  scale_color_discrete(name="Group",labels=c("group 1", "group 2"))+
  ylim(1.1, 1.55)

png(filename="traj.png", res=300, width=2500,height=1080)
plots=plot_grid(a,b,c, nrow=1,rel_widths=c(0.3,0.3,0.43))
print(plots)
dev.off()
```

As an additional validation of these results, these significant
clusters were extracted as regions-of-interests and fitted in a linear
mixed effects model using another R package— lmerTest (Kuznetsova, Brockhoff, and Christensen
2017).

```
library(lmerTest)
```

Linear mixed effect testing the effect of session, group, and session
\* group interaction on the positive RFT clusters’ average thickness
value

```
lme.RFT=lmer(clustRFT~session+group+session*group+(1|participant_id),data =dat_beh_ses13 )
summary(lme.RFT)
```

```
## Linear mixed model fit by REML. t-tests use Satterthwaite's method [
## lmerModLmerTest]
## Formula: clustRFT ~ session + group + session * group + (1 | participant_id)
##    Data: dat_beh_ses13
## 
## REML criterion at convergence: -317.1
## 
## Scaled residuals: 
##      Min       1Q   Median       3Q      Max 
## -2.69862 -0.43221 -0.04002  0.42291  2.57082 
## 
## Random effects:
##  Groups         Name        Variance Std.Dev.
##  participant_id (Intercept) 0.004837 0.06955 
##  Residual                   0.000236 0.01536 
## Number of obs: 96, groups:  participant_id, 48
## 
## Fixed effects:
##                Estimate Std. Error        df t value Pr(>|t|)    
## (Intercept)    1.326760   0.010717 54.685962 123.801  < 2e-16 ***
## session       -0.003450   0.001580 46.000000  -2.183   0.0342 *  
## group         -0.006877   0.010717 54.685962  -0.642   0.5237    
## session:group  0.007645   0.001580 46.000000   4.837 1.51e-05 ***
## ---
## Signif. codes:  0 '***' 0.001 '**' 0.01 '*' 0.05 '.' 0.1 ' ' 1
## 
## Correlation of Fixed Effects:
##             (Intr) sessin group 
## session     -0.295              
## group       -0.125  0.037       
## session:grp  0.037 -0.125 -0.295
```

Linear mixed effect testing the effect of session, group, and session
\* group interaction on the positive TFCE clusters’ average thickness
value

```
lme.posTFCE=lmer(clustCTTFCE~session+group+session*group+(1|participant_id),data =dat_beh_ses13 )
summary(lme.posTFCE)
```

```
## Linear mixed model fit by REML. t-tests use Satterthwaite's method [
## lmerModLmerTest]
## Formula: clustCTTFCE ~ session + group + session * group + (1 | participant_id)
##    Data: dat_beh_ses13
## 
## REML criterion at convergence: -361.8
## 
## Scaled residuals: 
##      Min       1Q   Median       3Q      Max 
## -2.78708 -0.33106  0.04481  0.40065  2.56232 
## 
## Random effects:
##  Groups         Name        Variance  Std.Dev.
##  participant_id (Intercept) 0.0035320 0.05943 
##  Residual                   0.0001231 0.01110 
## Number of obs: 96, groups:  participant_id, 48
## 
## Fixed effects:
##                Estimate Std. Error        df t value Pr(>|t|)    
## (Intercept)    1.317589   0.009015 52.274753 146.160  < 2e-16 ***
## session       -0.001788   0.001141 46.000000  -1.567    0.124    
## group         -0.006276   0.009015 52.274753  -0.696    0.489    
## session:group  0.005712   0.001141 46.000000   5.004 8.67e-06 ***
## ---
## Signif. codes:  0 '***' 0.001 '**' 0.01 '*' 0.05 '.' 0.1 ' ' 1
## 
## Correlation of Fixed Effects:
##             (Intr) sessin group 
## session     -0.253              
## group       -0.125  0.032       
## session:grp  0.032 -0.125 -0.253
```

Linear mixed effect testing the effect of session, group, and session
\* group interaction on the negative TFCE clusters’ average thickness
value

```
lme.negTFCE=lmer(neg.clustCTTFCE~session+group+session*group+(1|participant_id),data =dat_beh_ses13 )
summary(lme.negTFCE)
```

```
## Linear mixed model fit by REML. t-tests use Satterthwaite's method [
## lmerModLmerTest]
## Formula: 
## neg.clustCTTFCE ~ session + group + session * group + (1 | participant_id)
##    Data: dat_beh_ses13
## 
## REML criterion at convergence: -300.7
## 
## Scaled residuals: 
##      Min       1Q   Median       3Q      Max 
## -1.86716 -0.43759 -0.05507  0.46628  2.22097 
## 
## Random effects:
##  Groups         Name        Variance  Std.Dev.
##  participant_id (Intercept) 0.0045335 0.06733 
##  Residual                   0.0003547 0.01883 
## Number of obs: 96, groups:  participant_id, 48
## 
## Fixed effects:
##                 Estimate Std. Error         df t value Pr(>|t|)    
## (Intercept)    1.252e+00  1.071e-02  5.955e+01 116.848  < 2e-16 ***
## session        7.503e-06  1.937e-03  4.600e+01   0.004 0.996927    
## group          1.703e-02  1.071e-02  5.955e+01   1.590 0.117069    
## session:group -7.101e-03  1.937e-03  4.600e+01  -3.665 0.000638 ***
## ---
## Signif. codes:  0 '***' 0.001 '**' 0.01 '*' 0.05 '.' 0.1 ' ' 1
## 
## Correlation of Fixed Effects:
##             (Intr) sessin group 
## session     -0.362              
## group       -0.125  0.045       
## session:grp  0.045 -0.125 -0.362
```

## References:

Fink, Andreas, Karl Koschutnig, Thomas Zussner, Corinna M.
Perchtold-Stefan, Christian Rominger, Mathias Benedek, and Ilona
Papousek. 2021. “A Two-Week Running Intervention Reduces Symptoms
Related to Depression and Increases Hippocampal Volume in Young
Adults.” *Cortex* 144 (November): 70–81. https://doi.org/10.1016/j.cortex.2021.08.010.

Kuznetsova, Alexandra, Per B. Brockhoff, and Rune H. B. Christensen.
2017. “lmerTest Package:
Tests in Linear Mixed
Effects Models.” *Journal of
Statistical Software* 82 (December): 1–26. https://doi.org/10.18637/jss.v082.i13.
