# Supplementary material for: VertexWiseR: A package for simplified vertex-wise analyses of whole-brain and hippocampal surfaces in R
Source: Imaging Neurosci (Camb). 2024 Nov 14;2:imag-2-00372. doi: 10.1162/imag_a_00372 (PMC12330379; doi:10.1162/imag_a_00372)
Supplement: Supplementary Material 1 [file imag_a_00372-supp1.zip › imag_a_00372-supp3.pdf]

S3. Running the model from Example 1 with SPM's Computational Anatomy Toolbox (CAT12)

To replicate the example 1 analysis with the Spreng dataset, another model testing age effect on thickness, controlling for sex, was run in the same cohort using the Computational Anatomy Toolbox (CAT12) toolbox in MATLAB 2022b (Gaser et al., 2024). The pipeline consisted first in volume preprocessing with tissue segmentation of the T1 volumes, skull-stripping, and normalisation to MNI152 space. Cortical surface and thickness estimation was processed using projection-based thickness method (Dahnke et al., 2013) and the estimated surfaces registered to the fsaverage template. Just like with VertexWiseR, thickness was used as the measure of interest and spatial smoothing done with 10 mm FWHM. As in the example 1 analysis, the model tested the effect of age on cortical thickness, controlling for sex, with TFCE correction (with the same extent and height parameters and 1000 permutations). Supplemental figure 1 depicts the p-values within the clusters identified in each hemisphere, similarly large to Example 1. Likewise, only negative clusters were detected.

Supplemental figure 1. Effect of age on cortical thickness controlling for age, with threshold-free cluster enhancement in CAT12

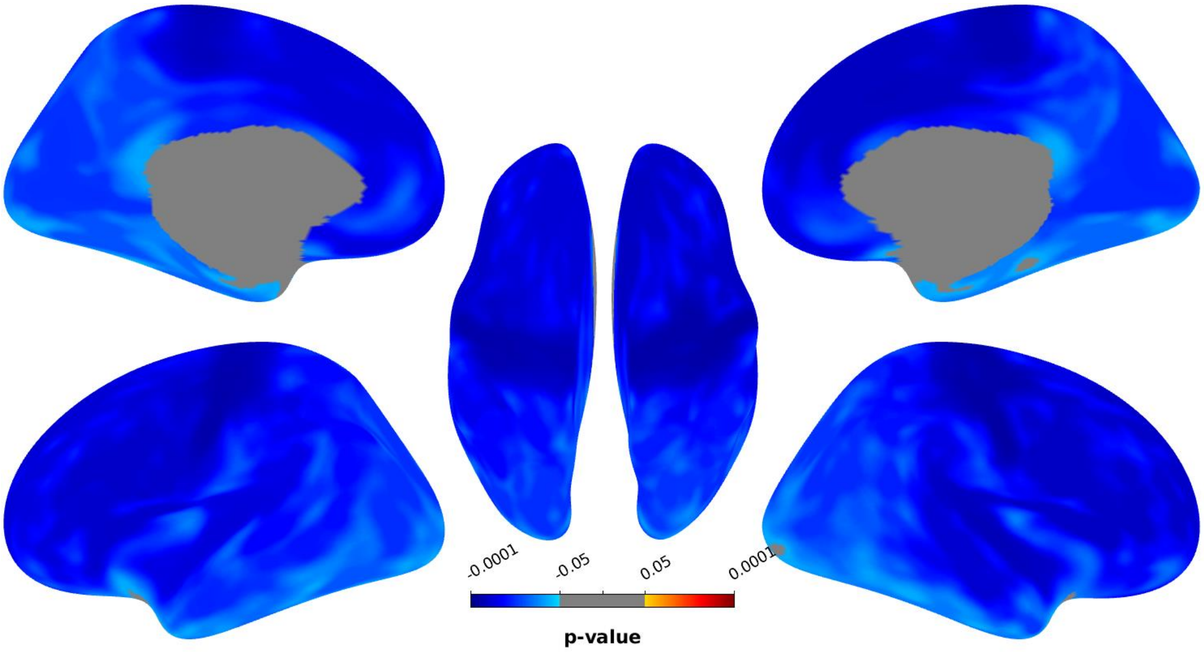

References:

Dahnke, R., Yotter, R. A., & Gaser, C. (2013). Cortical thickness and central surface estimation. *NeuroImage*, *65*, 336–348. https://doi.org/10.1016/j.neuroimage.2012.09.050

Gaser, C., Dahnke, R., Thompson, P. M., Kurth, F., Luders, E., & the Alzheimer's Disease Neuroimaging Initiative. (2024). CAT: A computational anatomy toolbox for the analysis of structural MRI data. *GigaScience*, *13*, giae049. https://doi.org/10.1093/gigascience/giae049
